# Calculating the Cost and Financing Needs of the Basic Package of Health Services in Afghanistan: Methods, Experiences, and Results

**DOI:** 10.9745/GHSP-D-21-00658

**Published:** 2022-08-30

**Authors:** Khwaja Mir Ahad Saeed, Salma Osmani, David Collins

**Affiliations:** a Independent, Kabul, Islamic Republic of Afghanistan; formerly of the Ministry of Public Health, Kabul, Islamic Republic of Afghanistan.; b Boston University School of Public Health, Boston, MA, USA.

## Abstract

The Basic Package of Health Services (BPHS) is the basis for primary health care in Afghanistan and should be accessible to all citizens. Most of these health services have been provided by nongovernmental organizations with support from donors. Studies before 2018 found that utilization and quality of services were lower than they should be, partly due to insufficient resources, leading the Ministry of Public Health to conduct a costing study to determine the level of funding required for the BPHS.

We expanded and refined that data analysis in this article. The main findings show that the total recurrent expenditure on BPHS interventions in 2018 was US$281 million (US$8.93 per capita)—only 62% of the US$452 million (US$14.34 per capita) required for good quality of care. It also showed that the need for services was probably not fully met by public facilities, with actual utilization less than 50% of the need in some cases. Furthermore, scaling up to entirely meet the need could require 2 to 3 times the resources used in 2018.

Following the change of government in 2021, economic problems, food shortages, reductions in donor funding, and other factors have increased the need for public health services while the capacity and quality of those services have deteriorated. Nongovernmental organizations continue to provide the BPHS, which remains the main platform for primary health care services in Afghanistan, and international organizations are working to rebuild and support these health services. But additional donor support is needed. The results of this study provide important information on the cost and financing needs of the BPHS that can be used for advocacy and for financing and planning services. We also describe the methodology, challenges, and solutions that can be helpful to other countries interested in conducting similar analyses.

## Abstract (precis)

We present a methodology for calculating the funds necessary to provide primary health care services and apply it to the Basic Package of Health Services in Afghanistan.

## Teaser

**Key Findings:**

- We show the 2018 cost and financing needs of the Basic Package of Health Services (BPHS) in Afghanistan based on modeling done by the Ministry of Public Health.
- The results indicate that the BPHS has been underfunded for the services it has provided, has not been reaching all the population in need, and would require significant additional financing for scale-up.

**Key Implications:**

- The lack of adequate financing for the BPHS has contributed to under-coverage, poor quality of care, and unaffordable out-of-pocket costs for many people.
- Dynamic modeling tools such as CORE Plus can help assess whether universal health coverage is being achieved and analyze gaps in financing.
- The resulting analysis should be used for advocacy and for financing and planning with the Taliban government and donors funding the BPHS.

## BACKGROUND

In 2003, the Afghanistan Ministry of Public Health (MOPH) developed a Basic Package of Health Services (BPHS) to provide a standard package of priority primary health care (PHC) services that would be accessible to all people, especially in underserved rural areas.^1^ In 2005, this was complemented by an Essential Package of Hospital Services^2^ to help increase referrals and access to inpatient services. The BPHS package has been provided by all the PHC facilities operated by the MOPH or by nongovernmental organizations (NGOs) under contract with the MOPH.

Implementation of the BPHS has increased access to and utilization of PHC services in rural areas; improved women’s access to basic health care; increased the number of births attended by skilled health personnel; and increased supplies of essential medicines.^3^ However, several studies have confirmed issues of poor quality and inefficiency.^4^^–^^7^ These include shortages of medicines so that patients receive no or incomplete treatment or have to buy medicine from private pharmacies, contributing to the high rate of catastrophic health expenditure.^5^^,^^6^^,^^8^ Shortages of supplies can also affect quality of care (e.g., a lack of laboratory tests or higher infection rates due to inadequate cleaning). Staffing shortages often mean longer waiting times, shorter consultations, and in some cases patients not seeing a provider at all. Due to cul-tural barriers, the lack of female health workers makes it even more difficult for women to access essential antenatal, obstetric, and postnatal care.^5^

A 2019 study also indicated access issues, noting that poorer people and people who lived in rural areas had not fared as well as others.^4^ Only 65% of lower-income people with illness accessed treatment outside their homes, compared with 80% of higher-income people, with the most common reasons being that services were too expensive and the belief that illness would go away by itself. Physical access to facilities was more limited for poorer and rural people, and poorer people were less satisfied with the quality of care. Although the BPHS was free of charge, many public medical facilities were not equipped with enough staff and medical supplies, and patients often had to buy drugs and supplies themselves.^5^ Private health services were expensive,^9^ and out-of-pocket costs were a significant financial burden for poorer families, particularly for medicines and supplies. Access challenges were not just due to geography but also to gender inequality, insecurity, and the costs of accessing health care.^10^ A 2020 assessment of the BPHS confirmed its achievements and recommended actions to improve efficiency, equity, and quality of care.^11^

To provide evidence of the need to increase funding, the Health Economics and Financing Directorate of the MOPH conducted a costing of the BPHS in 2016 and a follow-up costing in 2018.^12^ We conducted an additional analysis of the 2018 data to provide information that can be used to advocate for further financing, allocate funding and plan services in the current environment, and provide lessons on methods that can be useful for other countries. As we were writing this article, the Taliban came into power, and health care needs increased while health care services deteriorated.^13^ Health staff were sometimes not paid, female staff and patients began to face restrictions, and medicines and supplies have often been unavailable.^14^ There has been no opportunity for the MOPH to update the costing since the change of government, and we believe that the findings from this analysis based on 2018 data remain useful for the financing and planning of the BPHS.

Our analysis of the BPHS can be used to advocate for further financing, allocate and plan current funding, and provide lessons for other countries.

## METHODS

### Costing Tool

We calculated the recurrent cost of services actually provided and compared it with both the cost of the resources required to provide those services and the cost of the resources needed to scale up services. A tool called CORE Plus was selected for the costing because it was designed for that type of analysis.^15^^,^^16^ It is an activity-based health facility costing tool that was originally developed in Zimbabwe in 1995, has been widely used, and was included in an international review of costing tools.^17^ Actual costs are compared with actual numbers of services to provide average cost per service and per capita. Normative costs are calculated by combining the bottom-up calculation of direct costs based on standard treatment protocols, with indirect costs allocated based on technical staff minutes. These normative costs are used to calculate the required costs of actual services and the projected costs of scaled-up or modified services.

The tool produces reports for each of 3 main scenarios: current services and actual costs, current services and required costs, and needed services and required costs (Figure). The reports include total number of services broken down by type (e.g., curative) and program; total costs broken down by type, program, and resource type; lists of services ranked according to numbers of services or costs; quantification and cost of required medicines; and actual and required financing by source. The results show if the actual cost was appropriate for the current services and how many services and costs are needed to scale up services.

**FIGURE.**
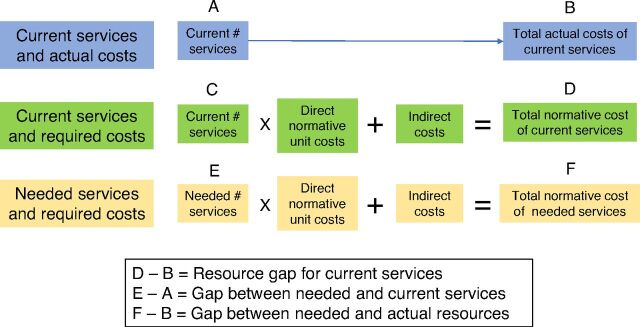
CORE Plus Costing Tool Scenarios to Calculate the Recurrent Cost of Primary Health Care Services in Afghanistan

### Data Collection

In 2018, the BPHS was provided by 309 mobile health teams (MHTs), 1,009 health subcenters (HSCs), 877 basic health centers (BHCs), 433 comprehensive health centers (CHCs), and 85 district hospitals (DHs) situated in 34 provinces. For simplicity, we describe all of these as facilities. Due to resource limitations, the sample of facilities was taken from 15 representative provinces and from 1 facility of each type in each province, selected based on the completeness of their health management information system quarterly reports. Using reporting completeness may have biased the sample toward better-resourced or better-managed facilities, but this was necessary for the costing. This resulted in a total sample of 67 health facilities (only 7 of the 15 provinces had MHTs). Community-based BPHS interventions provided through health posts were not included in the costing because they had been calculated previously.^18^

### Process

We first developed a list of BPHS interventions that matched with the indicators reported in the health information system and clearly defined each service so it could be costed. We included non-BPHS interventions provided by the facilities so that indirect costs would be fairly allocated. Data were collected for the year 2018—utilization data from the District Health Information System and expenditure data from the Expenditure Management Information System. A semistructured questionnaire was used to collect information from the facilities on topics including staffing and remuneration. It was not possible to obtain the actual cost of donated medicines and supplies (e.g., vaccines) so the normative costs were used based on the quantities provided. Only provider costs were included in the costing, and above-facility costs and capital costs were not included.

The needed numbers of services were based on the catchment population of each facility and incidence and prevalence rates developed by an expert panel of medical practitioners working in health facilities in Afghanistan, taking into account published data where available. These rates and their sources are shown in the Supplement. The figures were then reduced by the estimated proportion of the population that sought care in the private sector or in other public facilities and by the estimated proportion that had access limitations or did not seek treatment for minor ailments. Standard treatment protocols were used to calculate the normative direct unit cost for each service, comprising the type of health worker and the time needed, as well as the type and quantity of each medicine, supplies, and tests needed. These were developed by the expert panels based on national and international practices where available. Supply and lab test unit prices were based on data collected from implementer NGOs. Although the MOPH had standard remuneration rates, the actual rates paid were used in the costing because it was accepted that the NGOs would not be able to hire technical staff unless they paid these rates. No data existed for normative operating costs (e.g., administrative and support staffing, building repairs, and maintenance and utilities). In the absence of these data, we used the actual expenditures to represent the norms. The tool was used to prepare a separate CORE Plus model for each facility, and the results were combined to produce average national figures for each type of facility.

## RESULTS

### National

The average national figures presented are different from those shown in the report^8^ mainly due to the addition of donated medicines, vaccines, and nutrition supplies.

Table 1 shows the average catchment populations and utilization figures for the sampled facilities. Notably, the ranges across the facility types are wide—for example, some HSCs provided more services than some CHCs. The average actual catchment populations ranged from 6,561 for the HSCs to 59,237 for the DHs (see comments regarding reliability under Limitations). The total types of service provided were less than those specified in the BPHS, ranging from 57% of the package for the MHTs to 73% for the DHs. Several non-BPHS interventions were also provided and were included in the costing. The total number of services per capita, based on the total catchment population, ranged from 1.86 at the CHCs to 4.47 at the MHTs and may indicate problems with geographical access to higher-level facilities. The figure of 2.15 services per capita for the DH includes inpatient admissions. The majority of the services at all levels were preventive, ranging from 50.7% for the CHCs to 64.6% for the MHTs. The most utilized services at all levels were for child and adolescent health and development, ranging from 0.98 per capita at the CHCs to 3.16 at the MHTs (data not shown). Palliative care, rehabilitation, and surgical services were only provided at higher-level facilities. Diagnostic tests were not provided by MHTs, HSCs, or BHCs, so patients who needed such tests were treated symptomatically or referred to a CHC or DH.

**TABLE 1.** Average Population and Services Per Facility by Level of Care in 2018, Afghanistan

	**MHTs**	**HSCs**	**BHCs**	**CHCs**	**DHs**
Sampled number of facilities	7	15	15	15	15
Catchment population	7,500	6,561	13,201	30,123	59,237
Range	1,500–12,062	4,320–11,662	5,117–37,590	12,376–49,912	8,182–153,000
Types of service in BPHS package	79	81	83	86	155
Types of BPHS services provided	45	55	56	62	113
Range	42–47	49–61	50–65	57–66	99–130
Non-BPHS services provided	6	2	4	6	6
Range	5–7	1–3	2–5	4–7	4–8
Percentage of BPHS services provided	57	68	67	72	73
Range	53–59	60–75	60–78	66–76	63–83
Number of BPHS services	29,920	18,840	28,547	51,876	125,518
Range	13,794–67,259	5,124–38,207	15,985–46,478	22,828–124,938	39,181–226,515
Number of non-BPHS services	3,623	132	2,200	4,201	1,745
Total number of services	33,542	18,971	30,747	56,077	127,264
Total BPHS services per capita	3.99	2.87	2.16	1.72	2.12
Total non-BPHS services per capita	0.48	0.02	0.17	0.14	0.03
Total services per capita	4.47	2.89	2.33	1.86	2.15
Range	1.1–7.6	0.7–8.0	1.2–5.5	0.7–3.7	0.5–5.3

Abbreviations: BPHS, Basic Package of Health Services; BHC, basic health center; CHC, comprehensive health center; DH, district hospital; HSC, health sub-center; MHT, mobile health team.

The total average expenditure per facility, including donated medicines, vaccines, and nutrition supplies, was lowest at the HSCs (US$63,391) and highest at DHs (US$433,648) (Table 2). Differences across the facilities were largely due to varying service utilization levels and mixes and to different staffing numbers and remuneration levels. In addition, the DH package was larger, including more secondary level and inpatient services, and only the CHCs and DHs had laboratories. The average total actual expenditure per service ranged from US$3.15 for the BHCs to US$5.42 for the MHTs. The average expenditure per capita for the BPHS interventions ranged from US$5.43 for the CHCs to US$13.60 for the MHTs. The highest cost inputs were medicines and supplies, ranging from 42% of total costs at the DHs to 83% at the MHTs (including high-cost nutrition supplies). Technical staff salaries were the next highest cost component. Donated medicines, vaccines, and food ranged from 28% of total medicines and supplies costs for the DHs to 93% for the MHTs (data not shown). Most of the donations were vaccines, followed by nutrition supplies and TB medicines.

**TABLE 2.** Average Actual and Required Expenditure for Services by Level of Care in 2018, Afghanistan

	**MHTs**	**HSCs**	**BHCs**	**CHCs**	**DHs**
Catchment population	7,500	6,561	13,201	30,123	59,237
Number of BPHS services	29,920	18,840	28,547	51,876	125,518
Number of non-BPHS services	3,623	132	2,200	4,201	1,745
Total number of services	33,542	18,971	30,747	56,077	127,264
Actual total BPHS expenditure, US$	102,031	63,103	93,632	163,483	393,478
Actual total non-BPHS expenditure, US$	79,843	288	3,135	15,970	40,170
Actual total expenditure, US$	181,874	63,391	96,767	179,453	433,648
Actual required BPHS expenditure, US$	112,646	73,776	138,878	329,353	921,816
Actual required non-BPHS expenditure, US$	82,131	1,143	18,279	107,457	239,843
Actual required expenditure, US$	194,777	74,919	157,157	436,810	1,161,659
Actual cost per service, US$	5.42	3.34	3.15	3.20	3.41
Required cost per service, US$	5.81	3.95	5.11	7.79	9.13
Actual cost per capita of BPHS, US$	13.60	9.62	7.09	5.43	6.64
Required cost per capita of BPHS, US$	15.02	11.25	10.52	10.93	15.56
Actual cost per capita of non-BPHS services, US$	10.65	0.04	0.24	0.53	0.68
Required cost per capita of additional services, US$	10.95	0.17	1.38	3.57	4.05
Actual percentage of required cost for all services, %	93	85	62	41	37
Actual number of technical staff	4	4	5	11	30
Required number of technical staff	6	6	12	21	24

Abbreviations: BPHS, Basic Package of Health Services; BHC, basic health center; CHC, comprehensive health center; DH, district hospital; HSC, health sub-center; MHT, mobile health team.

Comparisons of the actual and required expenditures indicate that the services were under-resourced. The greatest shortfalls occurred at the DHs and CHCs, where only 37% and 41% of total needed resources were funded, respectively (Table 2). For example, the CHC spent US$5.43 per capita on BPHS interventions and US$0.53 for additional interventions but required US$10.93 per capita for the BPHS interventions and US$3.57 for additional interventions. All facility types were short of technical staff, except for the DHs which had too many, although this depends partly on the accuracy of the staffing component of the DH standard treatment protocols, which is more complicated for inpatient services. Although the MHTs needed 6 technical staff instead of 4, the number of staff may be limited due to the carrying capacity of the vehicles used. The DHs and CHCs had the greatest shortages of medicines and supplies, only spending 19% and 31%, respectively, of the amount required, which means that most patients did not get the quantities of medicines that they needed or had to buy them from private pharmacies (data not shown). Norms were not available for support staffing or facility operating costs and actual costs were used, so it is not known if these elements were under-resourced. The cost of the non-BPHS services was higher at the MHTs due mainly to the donated nutrition supplies for pregnant women and at the CHCs and DHs due partly to the cost of managing severe acute asthma and chronic obstructive pulmonary disease (COPD). These costs are estimates because of assumptions made in the allocation of indirect costs.

Comparisons of the actual and required expenditures indicate that the services were under-resourced.

Extrapolated from the sample, the total actual national utilization rate was 2.74 services per capita for BPHS interventions, based on the national population of 31.5 million (Table 3).^19^ The total recurrent expenditure was US$281 million (US$8.93 per capita), and the total required recurrent expenditure was US$452 million (US$14.34 per capita). The total actual recurrent BPHS expenditure was 62% of the required expenditure. The total actual national utilization rate (BPHS and non-BPHS interventions) was 2.9 services per capita, the total actual recurrent expenditure was US$319 million (US$10.14 per capita), and the total required recurrent expenditure was US$561 million (US$17.81 per capita). The total actual required recurrent expenditure was 57% of the required expenditure. Figures were based on the average cost of each facility and the number of each type of facility and included donated medicines and supplies. In terms of the required expenditure, 57% was needed for curative services and 41% for preventive services. The child and adolescent health program required 59% of the resources, followed by reproductive, maternal, and newborn health (17%) and chronic noncommunicable diseases (10%). Donated vaccines, nutrition supplies, and medicines comprised 47% of actual total national expenditure and 27% of required total national expenditure in 2018, indicating that the reliance on such donations was high (data not shown).

**TABLE 3.** Afghanistan National BPHS Costs Including Donated Medicines and Supplies in 2018

	**MHT**	**HSC**	**BHC**	**CHC**	**DH**	**Total**
Number of facilities	309	1,009	877	433	85	
Population	31,526,403	31,526,403	31,526,403	31,526,403	31,526,403	31,526,403
Number of BPHS services	9,245,148	19,009,294	25,035,427	22,462,452	10,669,062	86,421,383
Number of non-BPHS services	1,119,385	132,859	1,929,517	1,819,033	148,348	5,149,143
Total number of services	10,364,533	19,142,153	26,964,944	24,281,485	10,817,410	91,570,525
Average actual BPHS expenditure, US$	102,031	63,103	93,632	163,483	393,478	
Total actual BPHS expenditure	31,527,546	63,670,750	82,115,485	70,788,094	33,445,637	281,547,511
Average actual total expenditure, US$	181,874	63,391	96,767	179,453	433,648	
Total all actual expenditure, US$	56,198,962	63,961,736	84,864,581	77,703,151	36,860,122	319,588,552
Average required BPHS expenditure, US$	112,646	73,776	138,878	329,353	921,816	
Total required BPHS expenditure, US$	34,807,723	74,440,032	121,796,105	142,609,747	78,354,352	452,007,959
Average required total expenditure, US$	194,777	74,919	157,157	436,810	1,161,659	2,025,323
Total all required expenditure, US$	60,186,137	75,593,546	137,827,110	189,138,736	98,741,024	561,486,553
Per capita actual BPHS expenditure, US$	1.00	2.02	2.60	2.25	1.06	8.93
Per capita required BPHS expenditure, US$	1.10	2.36	3.86	4.52	2.49	14.34
Actual as percentage of required BPHS expenditure, US$	91	86	67	50	43	62
Per capita BPHS utilization, US$	0.29	0.60	0.79	0.71	0.34	2.74
Per capita actual total expenditure, US$	1.78	2.03	2.69	2.46	1.17	10.14
Per capita required total expenditure, US$	1.91	2.40	4.37	6.00	3.13	17.81
Actual percentage of required total expenditure, %	93	85	62	41	37	57
Per capita total utilization, US$	0.33	0.61	0.86	0.77	0.34	2.90

Abbreviations: BPHS, Basic Package of Health Services; BHC, basic health center; CHC, comprehensive health center; DH, district hospital; HSC, health sub-center; MHT, mobile health team.

Capital expenditure was not included in the figures, but according to the Expenditure Management Information System, a total of US$14 million was spent on these facilities in 2018, and the required capital expenditure was estimated at US$40 million. The national expenditure on community health services was also not included in the figures and was estimated at US$10.9 million (US$0.30 per capita) in 2018, based on a separate 2016 study,^18^ while the cost of the resources required to provide these services was US$32 million.

### Dykundi Province

A more in-depth analysis was done for 3 of the 5 nationally sampled facilities from Dykundi Province. The MHT and DH were excluded from the analysis because the ability of these facilities to improve and expand their services was limited by capacity constraints.

In 2018, the HSC, BHC, and CHC provided 70%, 71%, and 78%, respectively, of the BPHS interventions (Table 4). They also provided 2, 4, and 6 non-BPHS interventions, respectively. The total number of services utilized per capita was 1.4, 3.6, and 2.0, respectively. The actual expenditure per capita was US$5.25 for the HSC, US$12.76 for the BHC, and US$8.35 for the CHC compared with the required expenditure of US$6.47, US$19.73, and US$18.05, respectively.

**TABLE 4.** Cost Comparisons for Actual and Optimal Service Utilization for Sampled Dykundi Health Centers in 2018, Afghanistan

	**HSC**	**BHC**	**CHC**
Catchment population	8,275	5,117	21,942
Types of service in BPHS package	79	79	80
Types of BPHS services provided	55	56	62
Percentage of BPHS services provided	70	71	78
Types of non-BPHS services provided	2	4	6
Total number of actual services utilized	11,811	18,230	43,373
Total optimal number of services utilized	28,681	17,173	94,042
Number of services per capita actually utilized	1.4	3.6	2.0
Optimal number of services per capita	3.5	3.4	4.3
Actual expenditure, US$	43,470	65,272	183,210
Required expenditure, US$	53,539	100,946	396,115
Required expenditure for optimal number of services, US$	128,674	95,955	610,434
Actual expenditure per capita, US$	5.25	12.76	8.35
Required expenditure per capita, US$	6.47	19.73	18.05
Required expenditure per capita for optimal number of services, US$	15.55	18.75	27.82
Actual number of technical staff	3	5	11
Required number of technical staff	4	6	18
Required number of technical staff for optimal number of services	8	6	27
Actual percentage of optimal number of services, %	41	106	46
Actual percentage of required expenditure, %	81	65	46
Actual percentage of required expenditure for optimal services, %	34	68	30

Abbreviation: BPHS, Basic Package of Health Services; BHC, basic health center; CHC, comprehensive health center; HSC, health sub-center.

Optimal utilization levels for the full BPHS were set to calculate the cost of achieving universal health coverage. These levels were based on estimated incidence and prevalence rates for curative services and national targets for preventive services. The rates were then reduced to take into account the assumption that some of the population would use private or other public facilities (by 20% for the HSC, 25% for the BHC, and 15% for the CHC), and then by a further 20% for persons who would not be able or willing to access services (e.g., those choosing self-treatment for minor ailments). These figures are compatible with the findings of the 2018 Afghanistan Health Survey,^4^ taking into account that most, if not all, preventive services were provided by public facilities, incomes are low, and private sector facilities are few in Dykundi. Based on their catchment populations, the full package of services, and the optimal utilization levels, the utilization of these facilities should have been 3.5, 3.4, and 4.3 services per capita, respectively, more than double the actual rates for the HSC and CHC. And the required expenditure figures per capita for these optimal numbers of services would have been US$15.55, US$18.75, and US$27.82, respectively. The additional staff needed to provide the optimal numbers of services would have been 4 nurses and 1 midwife for the HSC, 2 additional nurses and 1 fewer vaccinator for the BHC, and 16 staff (mainly doctors and nurses) for the CHC. All of the expenditure figures included donated medicines and supplies and the actual remuneration rates for technical staff.

The ranking of the 2018 services by total required expenditure for the Dykundi CHC indicates that, if all the required resources were provided, the management of severe acute asthma and COPD would have represented 16% of total costs with US$62,756 (US$2.86 per capita) (Table 5). It should be noted that this is 1 of the additional services and not part of the BPHS. Urinary tract infections had the highest number of services but were only ranked fourth because the normative unit cost was relatively low at US$10.34. These ranking reports help to show where services might be prioritized and efficiency savings focused.

**TABLE 5.** Top 10 Actual Services at Dykundi CHC Ranked by Total Normative Cost for 2018, Afghanistan

**Service**	**Number of Services (%)**	**Total Cost, US$ (%)**	**Average Cost Per Service, US$**	**Average Cost Per Capita, US$**
Severe acute asthma and COPD	600 (1.4)	62,756 (16.0)	104.59	2.86
Severe acute malnutrition in children aged younger than 5 years	380 (0.9)	40,165 (10.0)	105.70	1.83
Early childhood development	2,857 (6.6)	35,385 (9.0)	12.39	1.61
Urinary tract infection	3,218 (7.4)	33,282 (8.0)	10.34	1.52
First antenatal visit	754 (1.7)	27,836 (7.0)	36.92	1.27
Prehospital care	612 (1.4)	20,013 (5.0)	32.70	0.91
Normal delivery at facility	339 (0.8)	18,459 (5.0)	54.45	0.84
Peptic disorder	2,566 (5.9)	16,500 (4.0)	6.43	0.75
First postnatal visit	606 (1.4)	9,755 (2.0)	16.10	0.44
PCV 13 vaccination	1,606 (3.7)	8,943 (2.0)	5.57	0.41

Abbreviations: CHC, comprehensive health center; COPD, chronic obstructive pulmonary disease; PCV, pneumococcal conjugate vaccine.

The top 10 services based on the optimal numbers of services show a different picture because they are based on needed rather than actual numbers of services. The 3 services with the highest total cost would be management of severe asthma and COPD, pneumonia for people aged 5 and over, and prehospital care.

## DISCUSSION

### Challenges and Recommendations

It took a lot of time and effort to convert the BPHS into a list of costed services that match the District Health Information System indicators. We recommend doing this step as part of the development of the package of services. Expenditure and health management information system utilization data were sometimes incomplete and incorrect—more effort should be put into supervision and data audit. Incidence and prevalence rates were not available for all diseases and had to be estimated by the expert panel. We recommend the creation of accessible international repositories for incidence and prevalence rates and standard treatment protocols for all common illnesses so that the need and normative cost of services can be more easily and accurately developed. Finally, it is important to take into account the costs and benefits to households when considering priorities; for example, ensuring adequate quantities of medicines and supplies would reduce the financial burden for patients who have to buy them from private providers, often at high prices.

### Catchment Populations

A review of the methodology recommended that the normative catchment population for each facility type should have been used for the modeling, instead of the actual catchment population reported by the facility.^15^ Since the normative populations were greater than the actual populations for the BHCs, CHCs, and DHs, using those figures would have resulted in a lower cost per capita. But, at the same time, the utilization per capita would have been lower, indicating possible inadequate coverage of facilities or access problems. While both methods of counting the catchment population can be used for costing, given the difficult terrain in Afghanistan, we preferred to use the actual reported population to measure efficiency and for planning. It is important, however, to recognize that catchment populations can be difficult to measure, especially in places where some people use facilities outside their areas or where there are different facility levels within the same area. Calculating catchment populations for MHTs can be difficult because people from those villages may use a static facility on days when the MHT does not visit or for more complex problems. It is also challenging to calculate catchment populations for DHs since they can have different populations for primary outpatient, secondary outpatient, and inpatient services and can have overlapping outpatient populations with other facilities.

Catchment populations can be difficult to measure, especially where some people use facilities outside their areas or where there are different facility levels within the same area.

### Sensitivity Analysis

The main resource costs in the total actual and required national expenditures were medicines and supplies (63% and 69%) and technical salaries (26% and 24%). A 10% reduction in the cost of medicines and supplies would have reduced total actual expenditures by US$20 million (6% of total costs). A 10% increase in technical salaries would have increased total actual expenditure by US$8 million (2.5%). These estimates were simple measures based on changing 1 variable while holding others constant. For individual services, the 2 highest cost services in terms of actual numbers and required expenditure at Dykundi CHC were management of severe acute asthma and COPD and management of severe acute malnutrition for children aged younger than 5 years, which had high unit costs for medicines and supplies. The third highest was improving early childhood development, which has a lower unit cost per service but a high number of services. The 3 services represent 35% of the total cost, but a change in the unit cost would not have a significant effect on total costs.

In terms of optimal costs, the most important factor is the number of services, which is derived from the catchment population, incidence rate, and utilization rate. At Dykundi CHC a reduction in the catchment population of 10% would lower the total cost by 9.5% and a reduction in the utilization rate of 10% (from 80% to 72%) would lower the total cost by 9.6%. The highest cost service was the management of severe acute asthma and COPD with 17% of total costs. The key factor was the incidence rate, estimated at 7.4% of the total population. Reducing that figure by 50% to 3.7% would reduce the total cost by US$20,204 (3% of total Dykundi CHC costs).

### BPHS Cost Comparisons

It is challenging to compare the cost of health service packages across countries and by using international estimates due to differences in package design, incidence and utilization rates, efficiency levels, and resource prices. However, several cost estimates have been prepared for Afghanistan since the launch of the BPHS. The initial package was estimated at US$4.55 per capita and a later analysis found that actual costs ranged from US$4.30 to US$5.12 per capita.^20^ A more recent study estimated the cost per capita in 2015 as US$7.64, including off-budget costs,^21^ whereas the total actual BPHS expenditure calculated with this study was US$8.93 per capita, which is 17% higher. However, the required funding for the services provided was US$12.16 for the BPHS, which is 61% higher than was actually spent.

### Use of Results

Before the change of government in 2021, the results of the 2018 BPHS costing were used by the MOPH to advocate for additional funding for the BPHS; to identify cost per service for a national Pay for Performance scheme; to estimate disease costs for national health accounts; to use in actuarial analysis for planning the introduction of health insurance; to develop investment cases, advocacy, planning, and budgeting for programs such as reproductive, maternal, neonatal, and child health, mental health, and family planning; and to assess the cost of possible new service delivery platforms. The cost models have also been used to estimate the initial costs of the Integrated Package of Essential Health Services, which has been under development since 2017 to consolidate the BPHS and the Essential Package of Hospital Services.^22^ The unit costs derived during the earlier 2016 BPHS costing were used in a study to estimate the costs and impact of scaling up services and in a study on priority setting.^23^^,^^24^

Before the 2021 government change, the 2018 BPHS costing results were used by the MOPH in advocacy, cost estimation, planning, budgeting, and other activities.

The results have also been used after the Taliban took over the government. The BPHS is still the accepted package for PHC and, since November 2021, donor-funded procurement of these services has been managed by the World Health Organization and UNICEF. Information and results from this technical report are being used to help inform the costs for contract negotiations with the service providers.

Following the change in government, the BPHS remains the accepted package for PHC and is now managed by the World Health Organization and UNICEF.

### Limitations

Limitations of our analyses include the exclusion of test costs for some lower-level facilities with laboratories. There were also no norms for numbers of administrative staff or operating costs and these were not increased in relation to scaled-up numbers of services and technical staffing. We did not take into account the capacity of facilities to make improvements in quality of care or to scale up outpatient service numbers (e.g., numbers of consultation rooms), nor did we assess the bed capacity of the CHCs and DHs to see if it was possible to expand the numbers of bed days to increase the length of stays or to scale up the numbers of admissions. Further analysis of the services and costs of the MHTs would be important since their relatively high costs may still provide the best and most affordable access to care in remote areas. We were unable to collect information on referrals upward or downward, and it was noted in a separate study that the referral system does not always function well.^25^ Finally, we did not discount the costs to present values.

## CONCLUSIONS

With concerns about the coverage and quality of PHC services, the MOPH in Afghanistan conducted a costing of the BPHS in both 2016 and 2018. We conducted additional analyses of the 2018 data to refine and expand the estimates of costs and financing needs. The results show that the BPHS interventions provided in 2018 were underfunded and that the need for services was not fully met by public facilities. Scaling up to meet that need could require 2 to 3 times the resources used in 2018.

Since the government takeover by the Taliban in 2021, health needs have increased due to economic problems, food shortages, and other issues, while health services have deteriorated due partly to reductions in donor assistance and access and staffing issues. While the BPHS remains the platform for PHC in Afghanistan, additional financing is needed to expand and improve services and the results of this study should continue to be used for advocacy and for the planning and financing of services. The reduction in health system resources since the change of government necessitates a costing update, which can be accomplished using the costing tool and models discussed here. In addition, this tool and our methodology can be used by other countries for similar research.

## Supplementary Material

GHSP-D-21-00658-supplement.xlsx

## Notes

**Peer Reviewed**

**First published online:** August 17, 2022.
